# Resveratrol Inhibits Trophoblast Apoptosis through Oxidative Stress in Preeclampsia-Model Rats

**DOI:** 10.3390/molecules191220570

**Published:** 2014-12-09

**Authors:** Yanfen Zou, Qing Zuo, Shiyun Huang, Xiang Yu, Ziyan Jiang, Shan Zou, Mingsong Fan, Lizhou Sun

**Affiliations:** 1 Department of Obstetrics and Gynecology, The First Affiliated Hospital of Nanjing Medical University, 300 Guangzhou Road, Nanjing 210029, Jiangsu, China; E-Mails: zouyanfen2011@163.com (Y.Z.); zuoqing127@163.com (Q.Z.); huangshiyun7@163.com (S.H.); zyjiangchm@163.com (Z.J.); zoushan06@163.com (S.Z.); fanmingsong01@163.com (M.F.); 2 Department of General Surgery, The Second Affiliated Hospital of Nanjing Medical University, 121 Jiangjiayuan Road, Xiaguan District, Nanjing 210000, Jiangsu, China; E-Mail: yuxiang861120@163.com

**Keywords:** resveratrol, HTR-8/SVneo cells, oxidative stress, apoptosis

## Abstract

Resveratrol has been shown to be a therapeutic agent for cardiovascular disorders by maintaining a lower redox level *in vivo* through its anti-oxidant properties. Resveratrol can prevent cells from p53- and reactive oxygen species-dependent apoptosis induced by interleukin-1b. We identified an inhibitory effect of resveratrol against oxidative stress and apoptosis using the TUNEL assay in NG-Nitro-l-arginine methyl ester-induced preeclampsia in rats. To investigate a possible association between resveratrol and the apoptosis caused by oxidative stress *in vitro*, assays for superoxide dismutase and malondialdehyde as well as flow cytometric analyses were conducted in HTR-8/SVneo cells after hypoxic treatment with or without resveratrol for 24 h. These data suggest that resveratrol significantly opposes the effects of oxidative stress *in vivo* and *in vitro*.

## 1. Introduction

Preeclampsia (PE) is a pregnancy-specific disease with a worldwide prevalence of 7%–12% [1]. As one of the most common pathologic complications of pregnancy, it is a leading contributor to pregnancy-associated mortality, and can cause serious damage to the heart, brain, kidneys, and other organs. The underlying pathogenesis of PE is usually considered to be insufficient perfusion of the uterus and placenta from the mother, which contributes to the ischemic and hypoxic microenvironment of placental trophoblasts. Recent evidence suggests that placental oxidative stress might be involved in the pathophysiologic characteristics of PE [2]. Oxidative stress activates several signaling pathways that restore homeostasis but, in case of failure, apoptotic machinery might be activated [3]. Several studies have found an increased level of apoptosis of villous trophoblasts in PE [4].

Resveratrol (*trans*-3,4′,5-trihydroxystilbene) is a naturally occurring compound present in several types of fruits, with the most abundant source being the skin of grapes [5]. Resveratrol has been shown to be a potential protective agent against cancer, inflammatory lesions, diabetes mellitus, and cardiovascular abnormalities [6]. Its potent cardioprotective functions are perhaps the best known. The cardiovascular disorders that may benefit from therapeutic administration of resveratrol include hypertension, myocardial infarction, arrhythmias, hypertrophy, and atherosclerosis [7,8]. Resveratrol has been proposed to ameliorate cardiovascular disorders via its anti-oxidant properties [9] because it is known to be a scavenger of superoxide, hydroxyl radicals, and peroxynitrite [10].

Moreover, resveratrol blocks interleukin (IL)-1b-induced apoptosis of chondrocytes via activation of silent mating type information regulation 2 homolog-1 and caspase-3 [11]. The p53- and reactive oxygen species (ROS)-dependent apoptosis induced by IL-1b can also be significantly opposed by resveratrol [12]. Additionally, the potential of resveratrol to protect chondrocytes against sodium nitroprusside-induced apoptosis via scavenging of ROS has been investigated by Liang *et al.* [13]. However, whether the anti-apoptotic role of resveratrol in PE is through anti-oxidative stress remains to be elucidated.

We identified an oxidative-stress reaction in pregnant rats with hypertension and proteinuria *in vivo* that could be reversed significantly by resveratrol. The anti-oxidative role of resveratrol was investigated further by stimulating this process *in vitro*. Results showed that resveratrol inhibited the level of oxidative stress in a trophoblast line (HTR-8/SVneo) pretreated under hypoxia for 24 h, thereby verifying the potential role of resveratrol as a therapeutic agent in PE.

## 2. Results

### 2.1. Resveratrol Reverses Various Phenotypes in PE Rats

The indicators of blood pressure (BP), urine volume, and creatinine level were normal before treatment (data not shown). After treatment, BP, urine volume, creatinine level, litter size, pup weight, placenta weight, and external malformations for all three groups were measured (Table 1). Results suggested that the NG-Nitro-l-arginine methyl ester (L-NAME) (n = 10)-injected group exhibited significant increases in BP and levels of protein/creatinine compared with the control (n = 8) and L-NAME plus RE groups (n = 9), thereby demonstrating successful construction of a PE model using L-NAME and reversal of the role of resveratrol in PE phenotypes. Significant differences were not observed among the three groups with regard to litter size, external malformations, or birth weight of fetuses (Table 1).

**Table 1:** Parameters of pregnant rats after each treatment.

	Control (n = 8)	L-NAME (n = 10)	L-NAME + RE (n = 9)	P ^a^
SBP *, mmHg	124.39 ± 11.30	171.22 ± 10.41	130.07 ± 15.62	<0.05
Protein mg/mL * (24 h)	0.317	0.383	0.507	<0.05
Number of fetuses	12.6	11.1	10.9	>0.05
Fetus birth weight (g)	1.97	1.64	1.69	>0.05
Placental weight (g)	1.46	1.38	1.48	>0.05
External malformations	0 1	1	0	>0.05

Data are the mean ± standard deviation; ^a^ Obtained by one-way analysis of variance using SPSS v13.0; RE: resveratrol; SBP, systolic blood pressure. * *p <* 0.05.

### 2.2. Resveratrol Reverses the Oxidative-Stress Reaction in Pregnant Rats with Hypertension

**Figure 1:**
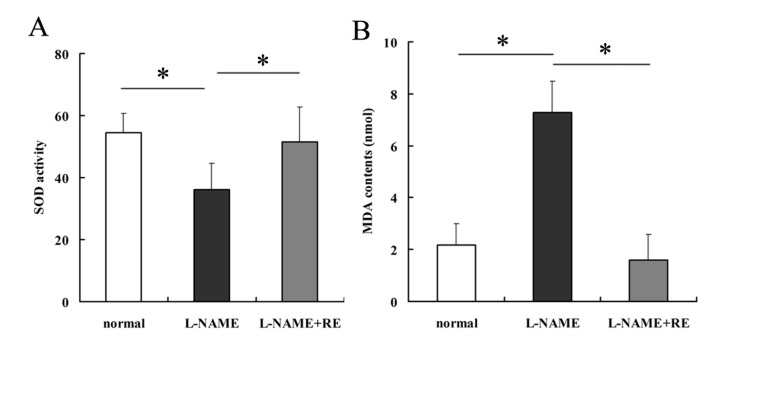

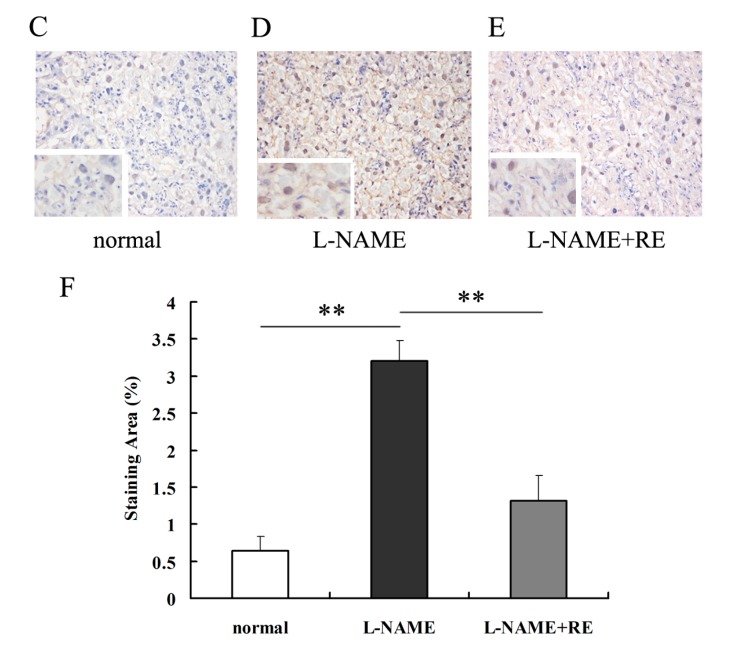
Assessment of oxidative states using assays of SOD and MDA, as well as apoptosis of placental cells in rats by the TUNEL assay. (**A**) SOD activity in rats injected with L-NAME exhibited a significant decrease compared with controls, whereas L-NAME plus RE groups appeared to reverse that trend. (**B**) MDA contents in rats injected with L-NAME were significantly higher than those of controls, and MDA contents in L-NAME plus RE groups were partly inhibited by resveratrol addition. (**C**–**F**) Placentas of L-NAME-injected rats showed a significant increase in apoptosis rate compared with those of normal pregnant and L-NAME plus RE groups as demonstrated by the TUNEL assay. Dark brown represents apoptotic cells and the blue denotes nuclei. Values are mean ± SD; * *p* < 0.05; ** *p* < 0.01.

Levels of the oxidant MDA were decreased whereas those of the antioxidant SOD were increased obviously in rats treated with L-NAME plus RE rather than those treated with L-NAME only (Figure 1A,B, *p* < 0.05). Both of these indices did not show a significant difference between control and L-NAME plus RE groups (*p* < 0.05). These findings provided evidence of the prevention of oxidative stress by resveratrol in PE.

### 2.3. Inhibition of Apoptosis by Resveratrol in PE Placentas

Apoptosis was more extensive in trophoblasts derived from the placentas of PE rats than in normal controls and resveratrol-treated rats as shown by the TUNEL assay (Figure 1C–E, *p* < 0.01). There was no significant difference between control and resveratrol-treated rats. These findings suggested that resveratrol had anti-apoptotic effects that acted through anti-oxidative-stress mechanisms. L-NAME induces damage to placental vessels by inhibiting eNOS expression as well as causing hypoxia and oxidative stress in trophoblasts: these effects could be reversed by resveratrol supplementation.

### 2.4. Resveratrol Inhibits Hypoxia-induced Oxidative Stress in HTR-8/SVneo Cells

**Figure 2:**
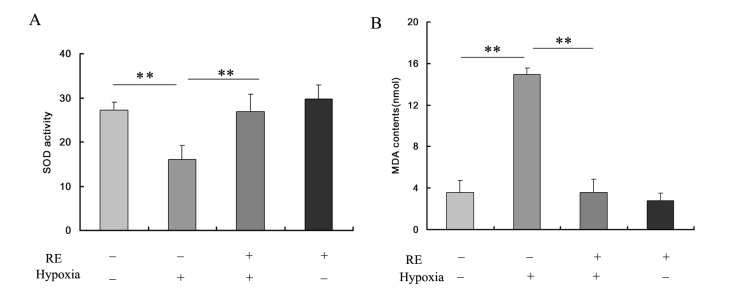
Assays for SOD and MDA in HTR-8/SVneo cells treated under hypoxia and resveratrol. (**A**) After culture in hypoxic conditions for 24 h, HTR-8/SVneo cells exhibited significant inhibition of SOD activity, and this inhibition was rescued by resveratrol supplementation. (**B**) MDA contents were increased after hypoxic culture and reversed by resveratrol. Values are mean ± SD; ** *p* < 0.01.

SOD expression was increased significantly in HTR-8/SVneo cells treated with 100 µmol/L resveratrol compared with 0 µmol/L resveratrol after induction of hypoxia for 24 h (Figure 2A,B, *p* < 0.01), whereas MDA expression decreased significantly (Figure 2A,B, *p* < 0.01). Cells treated with resveratrol alone showed the highest level of SOD and lowest level of MDA. SOD level was slightly higher in the resveratrol-only group than in control cells and the difference was not significant (Figure 2A,B, *p* > 0.05).

### 2.5. Resveratrol Decreases the Rate of Apoptosis of Trophoblasts Induced by Hypoxia

**Figure 3:**
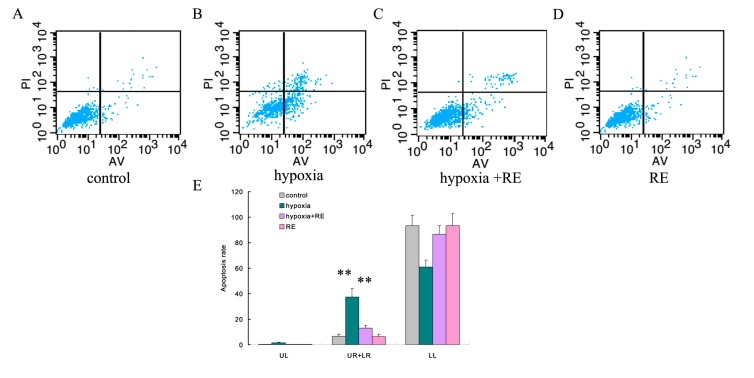
Rescue role of resveratrol in apoptosis of trophoblasts induced by hypoxia as detected by flow cytometry. (**A**) Apoptosis results of HTR-8/SVneo cells without any treatment (control). (**B**) Apoptosis of HTR-8/SVneo cells cultured in hypoxic conditions for 24 h showed a significant increase as compared with the control group. (**C**) Apoptosis of HTR-8/SVneo cells treated under hypoxic conditions and resveratrol revealed reversal of apoptosis compared with hypoxia-only treatment. (**D**) Cells treated with resveratrol alone displayed a similar level of apoptosis as those of control cells. (**E**) Statistical analyses of the flow cytometric data shown above (** *p <* 0.01).

Compared with the control, HTR-8/SVneo cells incubated in hypoxic conditions (Figure 3B) for 24 h had an increase in the rate of apoptosis as measured by flow cytometry (Figure 3A,B, *p* < 0.01). Reversal of apoptosis was detected when cells were supplemented with resveratrol as compared with the hypoxia group (Figure 3B,C, *p* < 0.01). Then resveratrol treatment alone is comparable to control conditions (Figure 3A,D, *p* > 0.05). Thus, the anti-apoptosis potential of resveratrol through anti-oxidative stress was verified *in vitro*.

## 3. Discussion

Resveratrol could induce cardiovascular protection and to have anti-inflammatory and anti-oxidant abilities. It can inhibit ROS generation [14], which could help to regulate apoptosis in several pathophysiologic and biologic processes [6]. The apoptosis-inhibitory effect of resveratrol as an anti-oxidant has also been reported by researches [11,12,13]. Studies have shown increased apoptosis in villous trophoblasts in pregnancies complicated by PE [4]. It is generally accepted that inadequate migration and invasion of placental trophoblasts into maternal spiral arteries could have a role in hypoxia of the placenta and impaired placentation [15]. Previously, we revealed specific proteins, such as C/EBP homologous protein (CHOP), in the endoplasmic reticulum stress pathway of apoptosis to be involved in endoplasmic reticulum-associated death in PE [16]. For several decades, efforts have been focused on elucidating the mechanisms of PE. However, the detailed mechanisms associated with apoptosis of trophoblasts remain unclear. With its anti-apoptosis function, resveratrol could have a protective role in the pathogenesis and development of PE.

Thus, in this present study, to explore the role of resveratrol in modulation of the apoptosis of trophoblasts in PE, *in vivo* and *in vitro* experiments were conducted. The TUNEL assay was employed to stain apoptotic cells in the placentas of PE-model rats. This assay showed a decreasing tendency in groups treated with resveratrol. Then, *in vivo*, detection of the activity of oxidative-stress indicators and flow cytometric analyses were employed to measure suppression of oxidative stress and apoptosis in HTR-8/SVneo cells by resveratrol. The SOD and MDA assays suggested that exogenous resveratrol prevents PE rats from undergoing apoptosis induced by oxidative-stress injury *in vivo* and *in vitro*. The connection between oxidative stress and apoptosis has been suggested by many reports [17,18]. SOD and MDA may not be sufficient to provide complete evidence for oxidative-stress, but these two indexes could partly reflect the level of ROS. Overproduction of ROS is able to cause cell damage through promoting DNA damage and regulation of apoptosis proteins [19].

On the other hand, the oxidative stress in PE could have been caused and rescued by other factors than resveratrol, resveratrol might have an anti-apoptotic role in trophoblasts in the pathogenesis of PE through mechanisms other than oxidative stress. Resveratrol has also been reported to contribute to other pathogenic processes that are apparent in PE. Melissa *et al.* found that resveratrol could inhibit the release of soluble fms-like tyrosine kinase-1 from human placentas [20]. Furthermore, resveratrol has a positive regulatory role on expression of endothelial nitric oxide synthase (eNOS) [21], as well as nitric oxide (NO) release [22], both of which are compromised in PE [23].

## 4. Experimental Section

### 4.1. Culture and Treatment of Cells

An immortalized, first-trimester, extravillous trophoblast (EVT) cell line, HTR-8/SVneo (kindly provided by Dr. Charles Graham, Queen’s University, Ontario, Canada), which was derived from a short-lived, primary EVT cell line, was used in the present study. HTR-8/SVneo cells were maintained in 1640 medium supplemented with 10% heat-inactivated fetal bovine serum, 100 U/mL penicillin, and 100 mg/mL streptomycin in standard culture conditions (37 °C in a 5%-humidified CO_2_ incubator).

Cells were separated randomly into four groups: control; hypoxia (24 h) plus RE (0 µmol/L); hypoxia (24 h) plus RE (100 µmol/L); normal oxygen plus RE (100 µmol/L). HTR-8/SVneo cells were seeded into six-well culture plates overnight and exposed to resveratrol (Selleck Chemicals, Houston, TX, USA) for 24 h.

For hypoxia experiments, HTR-8/SVneo cells after treatment with and without resveratrol were cultured under hypoxic conditions (1% O_2_, 5% CO_2_, and 94% N_2_) for 24 h.

### 4.2. Flow Cytometric Analyses of Apoptosis

After treatment for 48 h, HTR-8/SVneo cells were harvested using trypsin without ethylenediamine tetra-acetic acid. Then, they were washed with phosphate-buffered saline, resuspended in 1 mL binding buffer, and stained with fluorescein isothiocyanate–annexin V and propidium iodide at room temperature for 15 min in the dark according to the manufacturer’s recommendations. Cell analyses were by a Flow Cytometer (FACScan™; Becton Dickinson Biosciences, San Jose, CA, USA) equipped with Cell Quest™ software (Becton Dickinson Biosciences). Cells were sorted into living, necrotic, early-apoptotic, and late-apoptotic fractions. The relative ratio of early and late apoptotic cells was calculated for further comparison.

### 4.3. Assays for Superoxide Dismutase (SOD) and Malondialdehyde (MDA)

Media of HTR-8/SVneo cells after treatment (as mentioned above) were collected for detection of the oxidative stress index using a Total Superoxide Dismutase Assay kit (Beyotime, Beijing, China) for SOD, and a Lipid Peroxidation MDA Assay kit (Beyotime) for MDA.

### 4.4. Terminal Deoxyribonucleotide Transferase-Mediated dUTP Nick-End Labeling (TUNEL) Assay

A TUNEL kit (Roche, Basel, Switzerland) was used to accurately detect early apoptotic cells in rat placentas according to the manufacturer’s instructions. In the presence of terminal deoxynucleotidyl transferase (TdT enzyme), biotin-labeled 2′-deoxyuridine, 5′-triphosphate (dUTP) can link with the 3′-OH ends of the broken DNA of apoptotic cells. This connection binds specifically to streptavidin labeled by horseradish peroxidase and then reacts with hydrogen peroxide and diaminobenzidine to produce a dark-brown color, which can be observed under an optical microscope.

### 4.5. Animal Models

Animal experiments were approved by the Animal Care and Use Committee of Nanjing Medical University in accordance with the *Guidelines for use and care of animals* (National Institutes of Health, Bethesda, MD, USA). 

L-NAME was used to induce PE by intraperitoneal injection. As an eNOS inhibitor, L-NAME can cause dysfunction of vascular endothelial cells, and alter diastolic and systolic functions in arteries, which are similar to the clinical features observed in PE [24]. Several researchers regard these features, such as elevated blood pressure, increased proteinuria, and kidney injury, to be similar to PE seen in humans [25].

Mature, female Wistar albino rats (weighing 200–250 g), after confirmation of pregnancy, were divided immediately and randomly into three groups: control, L-NAME, and L-NAME plus RE. They were maintained in an air-conditioned room at an ambient temperature of 21 ± 3 °C and housed in separate cages.

L-NAME-injected rats were given 125 mg/kg body weight L-NAME from gestational day (GD) 0.5 to GD 18.5. Resveratrol (20 mg/kg per day) was given [26] by the intra-gastric route twice daily during the entire pregnancy. On DG 18.5, rats were killed and samples of urine, blood, and placenta obtained. Pups and placentas were dissected out and the litter size recorded. In addition, external malformations of all fetuses were examined. The fetuses and placentas were blotted dry and weighed.

BP was measured in all groups by the tail-cuff method using a Programmed Electro-sphygmomanometer (BP-98A; Softron, Tokyo, Japan) every day after pregnancy as described previously [27]. Urine was collected on GD 0.5, 4.5, 9, 13.5, and 18.5 of the pregnancy and stored at −80 °C. Urinary levels of albumin and creatinine were measured using kits (Beyotime).

### 4.6. Statistical Analyses

Data are the mean ± SD. Statistical analyses were carried out using SPSS v13 (IBM, Armonk, NY, USA). Paired and unpaired Student’s *t*-tests were used to compare results between two groups, and analysis of variance used to compare data from more than two groups. *p* < 0.05 was considered significant.

## 5. Conclusions

In conclusion, our results suggest the participation of resveratrol in the pathogenesis of PE and confirmed its therapeutic potential in PE. This is the first study to show the effects of resveratrol on oxidative stress and apoptosis in a rat model of PE and trophoblasts, respectively. However, the detailed downstream pathways and mechanisms involved in the contribution of resveratrol to PE are not known. Several studies have focused on the relationship between resveratrol and PE, but the clinical application of resveratrol in PE is a distant hope. Thus, a series of clinical trials with large cohorts will be required before resveratrol can be used to improve outcome in PE patients.
